# Exploring bias risks in artificial intelligence and targeted medicines manufacturing

**DOI:** 10.1186/s12910-024-01112-1

**Published:** 2024-10-17

**Authors:** Ngozi Nwebonyi, Francis McKay

**Affiliations:** 1 Department of Translational Health Sciences, Bristol Medical School, Learning and Research Building, University of Bristol, Level 1 Southmead Hospital, Bristol, BS10 5NB UK; 2 Population Health Sciences Institute, Newcastle University, Baddiley-Clark Bldg, Newcastle upon Tyne, NE2 4AX UK

**Keywords:** Bias, Artificial intelligence (AI), Targeted medicine manufacturing

## Abstract

**Background:**

Though artificial intelligence holds great value for healthcare, it may also amplify health inequalities through risks of bias. In this paper, we explore bias risks in targeted medicines manufacturing. Targeted medicines manufacturing refers to the act of making medicines targeted to individual patients or to subpopulations of patients within a general group, which can be achieved, for example, by means of cell and gene therapies. These manufacturing processes are increasingly reliant on digitalised systems which can be controlled by artificial intelligence algorithms. Whether and how bias might turn up in the process, however, is uncertain due to the novelty of the development.

**Methods:**

Examining stakeholder views across bioethics, precision medicine, and artificial intelligence, we document a range of opinions from eleven semi-structured interviews about the possibility of bias in AI-driven targeted therapies manufacturing.

**Result:**

Findings show that bias can emerge in upstream (research and development) and downstream (medicine production) processes when manufacturing targeted medicines. However, interviewees emphasized that downstream processes, particularly those not relying on patient or population data, may have lower bias risks. The study also identified a spectrum of bias meanings ranging from negative and ambivalent to positive and productive. Notably, some participants highlighted the potential for certain biases to have productive moral value in correcting health inequalities. This idea of “corrective bias” problematizes the conventional understanding of bias as primarily a negative concept defined by systematic error or unfair outcomes and suggests potential value in capitalizing on biases to help address health inequalities. Our analysis also indicates, however, that the concept of “corrective bias” requires further critical reflection before they can be used to this end.

**Supplementary Information:**

The online version contains supplementary material available at 10.1186/s12910-024-01112-1.

## Background

Artificial intelligence (AI) can be defined as a digital approach where algorithms are designed to perform human-like tasks by refining its operations as new data is incorporated into a learning database [1]. Analysts have stressed AI’s capacity to revolutionise healthcare, having substantial impact on such areas as medical diagnosis [1, 2], clinical trial design [3], and hospital management [4]. As healthcare becomes a data-intensive domain, AI can be mobilised to identify patterns in those data, enhancing the ways in which therapies are developed, governed, and delivered.

AI, however, may also amplify health inequalities through risks of bias [5–10]. This paper investigates the role of bias in AI driven targeted medicines manufacturing. Targeted medicines (also sometimes called precision medicines, personalised medicines, etc.) are medicines designed to target a particular patient within a given population or a subpopulation of patients within a general group. They are made possible by applying the principles and techniques of regenerative medicine, where the self-healing capacities of human bodies are potentialised by therapeutic interventions and cutting-edge technologies. Targeted medicines manufacturing refers to the act of making those medicines and can be separated into upstream and downstream processes, that is, into activities associated with research and development such as clinical trial design and data collection or analysis, or activities involved in producing the medicine in the laboratory, including the quality control processes and supply chain management that ensures a viable product gets to the patient. Targeted medicines manufacturing requires immense technological challenges to be overcome due to the variability of starting manufacturing materials (cells and tissues collected from patients), as well as the fragility of such materials, which can be destroyed or made unviable, if refined manufacturing processes are not mobilized [11, 12]. Insofar as these manufacturing systems require intense data generation and processing associated with quality control, AI becomes a key ally [13, 14].

Biases can potentially emerge in either upstream or downstream processes. How exactly and to what degree, however, is uncertain due to the novelty of AI’s application to the field. We therefore undertake here an exploratory study of targeted medicine manufacturing focusing on the question of bias. Exploratory studies are preliminary forms of research done to scope the parameters of a project before more in-depth investigation can be undertaken or to discover new ideas in areas that have received little prior research [15]. Here we interview experts working in the fields of bioethics, targeted medicines, and artificial intelligence to better understand whether and how bias might turn up in the future of targeted medicines. Through those voices we document a range of views on the possibilities of bias spanning the negative or ambivalent on the one hand and the positive and productive on the other. Though the former are commonly discussed in the literature on bias, the latter are particularly noteworthy as they offer novel approaches for how to think about bias mitigation. This is because bias has generally been theorised in the medical sciences, social sciences and humanities either as systematic error or by the inequalities it produces [6, 7, 16–20]. As Pot, Kieusseyan, and Prainsack [16] say, biases are “systematic distortions of datasets, algorithms, or human decision making… [with] negative effects… in terms of accuracy, fairness, or transparency.” As some interviewees note, however, not all biases entail error or inequity. Rather, some biases might help readdress health inequalities, if appropriately developed and harnessed. Hence, we suggest that rather than focus solely on removing biases, targeted medicines manufacturing should take notice of, and further think through, the corrective potential of biases. However, we also note that the concept of “corrective bias” demands further critical reflection if it is to function appropriately for this end.

In arguing this, we first outline the methods of our study, followed by the evidence. Finally, in the discussion section, we critically reflect on the conceptualisations of bias given by our interviewees and ask what the concept of corrective bias might mean for the future of target medicines manufacturing.

## Methods

Our project began as an exploratory ethics study on the potential impacts of bias in digitalised targeted medicines manufacturing. Given the nascent development of AI-driven targeted medicines manufacturing and the lack of published information available on the topic vis-à-vis bias, the method of interviewing expert stakeholders was chosen on account that such persons would be best placed to comment on the future ethical horizons of the field. The research was undertaken over a period of six months, from January to June 2023, with two months given for data collection. Interviewees included researchers and industry specialists in the UK with overlapping expertise in targeted medicine manufacturing (*n* = 6), medical AI research (*n* = 6), and bioethics (*n* = 2). Ethics permission was granted by the University of Oxford Central University Research Ethics Committee (reference number: R79245_RE001).

We planned for a minimum of 10 interviews. We used the concept of data saturation, defined as the decreasing ability to induct new themes from given data, as a theoretical ideal for the research and to help justify the reasonableness of sample size prior to data collection [21]. Estimates suggest saturation can be reached between 6 and 12 interviews [22] and we chose 10 interviews, with opportunity for more if time allowed, as a midway point, due to the short timeframe of the research. The degree to which we achieved saturation in the analysis stage is taken up further in the discussion section.

Twenty-two email invites were sent out with the study information leaflets to potential participants. One person declined while ten did not respond despite email follow-up. The final sample yielded 11 interviewees, obtained via snowball sampling.^1^ The Interviews were conducted remotely via Microsoft Teams in March and April 2023 and lasted between thirty and sixty minutes. Interviews were semi-structured, allowing for flexibility in exploring emerging themes while ensuring consistency across interviews. Interview questions focused on participant’s understanding of core concepts and relevant issues such as targeted medicines, AI, the anticipatory risks of bias, and how such biases might be mitigated. The interviewer did not define key concepts but left it open for interviewees to use and define them as they wished. In general, interviews largely used the language of “precision medicine,” though the paper adopts the term “targeted medicines” to avoid misleading connotations of “precision,” which may imply a high level of accuracy to the medicines that some may not agree with.

Interviews were audio-recorded and pseudonymously transcribed. Data was thematically analysed and coded using NVivo. We employed a hybrid, iterative approach to coding, combining inductive and deductive methods [23]. The initial round of coding utilized a semi-structured approach, guided by broad categories derived from our interview guide. These categories served as an initial framework for identifying subthemes and included reference to general social biases (those applicable to multiple research phenomena, not just AI) and specific biases (those particular to AI technologies in targeted medicines manufacturing), as well as potential mitigation strategies for both types. This approach facilitated the identification of subcategories of bias as highlighted in the results section (e.g., demographic, geographic, and financial biases). We then conducted a second round of deductive coding, applying the subcodes generated in the first phase to ensure comprehensive coverage of the data. Each phase was coded independently by two researchers (FM and NN), who also discussed themes at the end of each phase to develop consensus on the codebook. The codes were then contrasted with themes in the AI ethics literature to derive novel insights by comparison.

## Results

### Social Biases in Globalised Biomanufacturing

Social bias is a central theme of AI ethics, where it refers to historical forms of discrimination against particular groups as embedded in technological systems [6, 10]. For the majority of our interviewees, it was this kind of bias that was the primary concern. Sandra (a medical AI ethics researcher, working on data diversity, AI fairness, and genetics), described the issue as a “structural” problem which was “deeply integrated into… the DNA of our society” and hence was inevitable in targeted medicines. In doing so, she echoed a theme that was voiced in some form by most interviewees.

Social biases have a human and technical component. On the one hand, they could be manifested psychologically, as implicit cognitive biases. Oscar (an industry specialist in supply chains for targeted medicines), was most concerned about this. “Subconsciously,” he said, “everyone is shaped by their environment” and so, “when humans make decisions, they are always subject to conscious or unconscious biases which influence their decision making.” On the other hand, they could be found in unrepresentative datasets or through AI models that overrepresent some populations, organisations, and countries to the detriment of others. Sandra (medical AI ethics), for instance, discussed the issue in terms of socioeconomic statuses, i.e., the overlapping of geographic, demographic, and economic variables, due to data records being more readily available in wealthier areas. This was a concern for her because she worried that people falling outside of these areas would be “less well-served” in data records. Other interviewees described it similarly, with location, ethnicity, and wealth being the main factors to consider.

#### Geographic and Demographic Biases in Data Collection

Several interviewees noted how data and manufacturing resources for targeted medicines were predominantly found in Western countries. One participant, Irene (a science and health innovation policy researcher working on targeted medicines), noted that targeted medicine datasets were “primarily drawn from Europe and America.” This was especially a concern for genomic data, which was “dominated by individuals of European ancestry.” One key cause for this geographic and demographic bias was the underdeveloped state of digitalisation within lower- and middle-income countries (LMICs). As Irene noted, in those countries “data may not be in electronic formats” but “in paper format” instead, making access difficult and thus underrepresentation from these regions more likely.

Where this was not the case, there were still obstacles in accessing data and thus of developing globally representative datasets. Kate (a medical AI ethics researcher focusing on health inequalities in data-driven systems), noted that the “training datasets that AI technologies use are often not diverse… [and] have big gaps in certain ethnicity groups… [or] people who have not accessed [healthcare].” Consequently, any AI models built using those datasets would be compromised. Greg (a researcher specialising in building AI models using digital twins for targeted medicine manufacturing), spoke similarly: “there is always going to be a bias with these models… [because] a lot of the times the data… [used] to generate these models has… not [been] necessarily representative of the wider population… if there is bias in the data, then there is obviously going to be bias in the model.”

For Irene (science and health innovation policy), that bias was not unique to AI, but deeply rooted in the research practices supporting data collection. As she said, “AI is [not] the only technology that suffers from bias” as it could be found in the “randomised control trials” supporting research. Systemic biases in research processes like these meant their applicability to AI was inevitable. In addition, however, she also recognised that unique challenges and questions needed to be addressed regarding data transferability and safety if future data is to be efficiently shared for the purposes of AI research. As she asked: “How will you share it? Who will get credit? What about consent? What about the political constraints between sharing data in between two countries?” All these needed answers if one were to maximise data representation and thereby mitigate the risk of geographical bias.

#### Economic Biases

A second major pathway for bias in targeted medicines is cost. On the one hand, there is the issue of access. “These medicines are extraordinarily expensive,” said Ed (an industry specialist in bioprocessing of targeted medicines). “Some of them are really too expensive for the NHS,” he noted, and can cost upwards of “three or four hundred thousand pounds”. As a result, access to them is not universal, but available to only a select “few people.” This opened the possibility of biases of affluence, he feared, with targeted medicines being available only to those with the means to pay for them. “It is only sort of well-off people in well-off countries that can access it,” he said, emphasizing the point.

Some interviewees did posit that AI might help reduce the high costs of precision medicines, by scaling production and optimizing manufacturing processes. However, participants were not clear on how this would prevent biases if data were unrepresentative in the first place. Moreover, where costs were not borne directly by the consumer or the manufacturer, there could still be bias in the decision-making process determining who gets access to medicines. Max (a researcher working on optimisation processes for targeted medicines manufacturing), explained the problem: “There are certain disabilities that people have, which can be alleviated by things like gene therapy, but it’s very expensive.” In the UK, usually, the National Institute for Health and Care Excellence [NICE] “makes a decision [about] whether or not to authorise [fund] a particular therapy…[and] that’s not a technical decision.” Rather, “it involves judgement about the value of extending the life of somebody and improving their quality of life versus the cost.”

#### Low risk of bias when using non-patient data in downstream medicine production processes

All the above represent credible risks for targeted medicines manufacturing. Some areas, however, are seen by our interviewees as having low or negligible risks of bias. This is the case, for instance, with uses of AI in supply chain optimisation (that is, the use of integrated data-driven systems to enhance the managements of supply chains). Oscar (industry specialist for targeted medicines), for instance, posited that bias might have little impact there since it did not rely on patient or population data. As he put it: “I don’t see AI in manufacturing and supply chain having any kind of bias … because you’re really removed from any kind of patient treatment scenario”. That said, he was keen to clarify that bias could apply elsewhere. “If you look beyond manufacturing and supply,” for instance, research and development, and “commercial strategies, you would have to think… there is bias in those bots, consciously and subconsciously.” A key reason, then, for anticipating low risk of bias when AI was used as a decisional tool to control manufacturing processes or supply chain optimization stemmed from the kind of data being used. Such data were not from or related to patients but were derived from “factory floor scheduling” records, that is, from the day-to-day running of machines in the factory (e.g., processing times, failure rates, etc.). These could be automated using AI algorithms to regulate and improve the speed of workflow in the manufacturing of targeted medicines. Whether social bias could turn up here is uncertain but thought unlikely.

In the case of allogeneic therapies (those produced with cells taken from people other than the targeted patient), risks of bias may be more pronounced, however, especially where application of digital twins is involved. Digital twins are virtual representation of objects, human body parts or cells, using real-time data, and they can be used to predict cell quality prior to manufacturing of personalised medicines like chimeric antigen receptor (CAR) T cell therapies. According to Greg (digital twins and targeted medicines), this process is modelled using data within the laboratory: “we… [grow] these cells in the bioreactor… using healthy donors.” However, “the cell-growth rate… [in] healthy donors would probably be different… to patient-derived cells.” So, “the predictions would be based off… healthy donors… [which might not] be representative of… patient cells.” AI models using digital twins as a predictive tool would thus exhibit higher risk of bias due to it implicating population, rather than patient, data, and by developing synthetic datasets that could be unrepresentative of the treatment population. Decoupling data sources from clinical targets could therefore generate new kinds of biases in the domain of cell and gene therapies.

### Mitigating Bias

Regarding the question of how to protect against these biases, respondents gave multiple views. Some, for instance, were unclear about how to address bias, and were even sceptical whether the field had sufficient forms of redress. “How do you control or mitigate bias?… I wish I had an answer for you, but unfortunately, I do not, and I doubt if anyone at this point does” (Irene, science and health innovation policy researcher). In general, however, respondents were optimistic that bespoke approaches might be developed, using both AI and non-AI solutions.

#### AI v non-AI Mitigation Strategies

Greg (digital twins and targeted medicines researcher), for instance, suggested that some AI might be self-correcting. As he put it, “I expect… that we [would] switch from models that we train once… to models that learn lifelong continuously.” That way, even if there is initially a bias stemming from a model being trained on unrepresentative data, as more cells came in, they would “follow the real distribution… and the model should self-correct”. Underrepresentation in this view would thus be mostly a temporary issue and would be corrected over time as more data was gathered.

Several of our interviewees, however, were sceptical about the possibility of using AI to overcome bias. Partly this was due to a perception that data bias was a perennial issue. “My view of targeted medicines is that it is inherently biased, because it depends on a sample of the population that is being looked at” and because “a lot of this bias… [is] already embedded within health technology development” (Irene, science and health innovation policy). It was also partly due to a scepticism over the power of AI to detect or correct for biases. “There needs to be an awareness that AI is not always better, or it is not always the solution,” said Kate (medical AI ethics researcher). “These technologies do not always have a remit to flag or mitigate health inequalities …often their remit is to be more efficient or streamline things.” Hence, “there is a risk that we… [try] to turn everything into an AI solution thinking that AI is always better… [But] it is not and it might not work that well if it does not have good data.” Kate here implicitly referenced a tension that Charles (an AI technology researcher working on optimisation processes of targeted medicine manufacturing), put explicitly, namely, the tension between accuracy and fairness: “With these models… you never really get something optimal… you have to potentially make a compromise between the accuracy of the model and the fairness of the model. And you would [have to] be willing to potentially sacrifice a little bit of accuracy and pick a model that is slightly less accurate but is fair and unbiased.” In that sense, the promise of an AI solution or the belief that AI might develop a better or more objective representation was itself a kind of bias for a technological fix, one that failed to recognise inherent trade-offs between research values like accuracy and equity.

#### Patient and Public Involvement

Overall, there was advocacy for exploring non-AI or non-technological solutions to mitigate bias. One crucial area highlighted was patient and public involvement (PPI). PPI was thought as essential prior to developing AI technologies to avoid biases resulting from unrepresentative datasets. As Kate (medical AI ethics researcher) said, “the answer to gaps in data is not necessarily to just collect more data on people and try and get people to share their data, because there is a step of trust that needs to happen before that.” Kate’s point was that data is sometimes unrepresentative not just because researchers overlook collecting diverse data, but because there may be reluctance from certain groups to share data in the first place and that this reluctance can impact research. “Often, the people who are designing and deploying health technologies do not have all the information they need about, for example, why people might not use a certain technology or why people might not get the same results as other people.” The advised solution would thus be to “involve [more] diverse groups in the… very start of the planning of technologies.” Doing so would help better build trust in data sharing and AI tools and thus ideally improve the diversity in datasets that such models would use.

#### Transparency in AI Datasets

Greater transparency was also said to be needed in AI datasets to further limit pathways for bias. Transparency here could involve the development of standards to inform data scientists on the limitations of the AI models built with such datasets. Zara, (a medical AI researcher focusing on data inequalities in AI), stated as much: “We do not have good summaries of datasets so that other people can make decisions or make interpretations about whether the dataset is likely to be biased or not. We need to build… [that]. Datasets that are transparent about their bias[es]… [are] much more valuable than… dataset[s] that seems to be unbiased but only because… [they are] not being transparent.” Building transparency into datasets meant, for Zara, providing metadata alongside the datasets and explicitly acknowledging features important to the dataset that might be relevant for making judgments regarding bias, such as the types of population it represents and at what proportion, the likelihood of bias and in what aspect, etc. This would help ensure that when AI models are built with those datasets, bias risks are clearer to perceive or anticipate. This, in turn, could guide on suitable target populations for whom the models can be deployed, or where more data might be needed to make the model more representative.

#### Corrective Bias: a Potential Tool for Addressing Inequalities

Finally, and perhaps unintuitively, it was also recognised that bias itself might be used to counter the effects of certain other types of bias risks. Counter to the view that bias is largely a negative issue warranting mitigation at every instance, here bias was suggested, sometimes implicitly, sometimes explicitly, to have value as a corrective against various kinds of research harms. Olivia (a life science industry expert focusing on targeted medicines manufacturing processes), clarified: “Sometimes bias is kind of necessary to mitigate risks.” She made the point by analogy, in relation to medical research in general. Pregnant women, she noted, are sometimes excluded from clinical trials to prevent harm to an unborn child. Though this can potentially lead to bias if pregnant women are excluded from the research, this exclusion is often deemed acceptable insofar as it protects potential harms to the mother or unborn child. She admitted that this is more likely to be done where the target condition is not prevalent in, or is anticipated to pose low risk to, pregnant women. Nevertheless, the point remained that this may be a kind of bias and one that may be permissible if the trade-off protects another value of equal or greater importance.

In the same way that biases might sometimes be used to mitigate research harms in general, they could also therefore be used to minimize the effects of other biases, by targeting groups otherwise underrepresented in datasets. Zara (medical AI and inequalities researcher) advocated, for instance, for targeted medicines “to increase participation or data contribution from underrepresented [groups].” Though she recognised that this was “a form of biased research,” it is done “intentionally… [and] for a good reason.” Such an approach was often employed for rare diseases, Kate (medical AI ethics researcher) noted, where there is a natural underrepresentation of a particular condition in research or a dataset. Here a putative bias favouring the inclusion of poorly represented data was “not a problem,” as “it [is] sort of like rebalancing… [it is] a positive bias because finally there are opportunities to treat neglected diseases.” The point was further emphasised by Greg (digital twins and targeted medicines expert): “You can develop more targeted drugs that will only basically work for a certain population, but an alternate benefit of this is… [that] those people could not be treated [before] because they were only a small population.”

Another instance of what one might call “corrective bias” was noted for genetic diseases that are not rare, but prevalent in ethnic minority populations. As Ed (Industry expert in targeted medicines) noted, “sickle cell anaemia is… mainly prevalent in India, and… Afro-Caribbean groups”. “There are [targeted] treatments for that”. Since, however, such groups tend to be “underrepresented” in terms of research and therefore treatment, targeting approaches for that disease was morally permissible. As Ed confirmed, “I am not seeing it as disadvantaging, because… people are seeing that things like sickle cell anaemia are important and are looking at ways of curing them. So, I am not seeing sort of discrimination or bias in that way.”

For Kate (medical AI ethics), the issue pointed to something more general about how the word bias is used in health care. As she said, “we always think about…” bias as “widening health inequalities.” However, an alternative understanding could be “targeting… people who were not well served before…” and using this bias “to rebalance” the inequality. Though bias is still a discrimination, the question is whether such discrimination creates harm or promotes injustice and inequity, not whether it is biased per se. If bias can be used to address an existing inequity or mitigate a potential risk, then that may be justifiable.

## Discussion

The findings of our exploratory study highlight a spectrum of concerns for where bias is anticipated in targeted medicines manufacturing. The most salient issue mentioned was social bias and how it might impact AI via unrepresentative datasets, with demographic, geographic and financial biases being singled out as particularly noteworthy. In many ways, this confirms existing literature on bias and AI or precision medicine, which recognizes how social biases, whether implicit or explicit, may enter medical research in a variety of ways: for instance, through cognitive biases of researchers, through framing of research paradigms, through unrepresentative data, etc [5, 8, 10, 24, 25]. In certain areas of downstream manufacturing processes, however, such as in supply chain optimisation, bias pathways were less certain, and the riskiness was largely dependent upon whether AI relied upon patient or population health data or not. Greater salience was thus given to upstream research and development processes and the contexts surrounding that, including how data representation, access to medicines, and healthcare priority setting might be pathways for bias. This emphasis is further reflected in the mitigation strategies (patient and public involvement, greater transparency around dataset creation, and use of positive or corrective biases to readdress absences in datasets) which applied largely to broader contextual issues with participants largely silent about downstream mitigation efforts.

The emphasis given to upstream bias risks may partly reflect the ambivalence of the term “manufacturing,” which can be interpreted in a narrow sense (as referring solely to medicine production processes) or in a broad sense (as everything that goes into the development of a medicine, including research and development and the contextual issues shaping that process). That the latter influenced perceptions of bias risks for our interviewees shows the interconnected nature of bias in AI and precision medicine. Partly, however, it may reflect the difficulty of specifying bias risks in downstream processes due to lack of understanding. Notably, our interviewees had expertise in different areas of bias, AI and precision medicine, meaning that those working on medicine production processes may have limited awareness of the kinds of issues that bioethicists are comfortable discussing (such as bias), whereas those interested primarily in ethical issues may lack technical understanding of medicine production to know where bias may turn up during this stage.

Because of the uncertainty around downstream manufacturing processes, there is a question of whether data saturation was achieved in the project. In our experience, themes concerned social bias emerged early in the interview process, particularly regarding demographic, geographic, and financial biases in AI, as well as mitigation strategies like public involvement and transparency. This is arguably expected given that these are common themes in the AI ethics literature and therefore most likely to be at the forefront of an interviewee’s imagination. Other offerings were arguably more novel, including acknowledging low bias risks in supply chain optimisation as well as the use of corrective biases as mitigation strategies. That interviewees could only articulate these issues to a limited degree, however, suggests that while we may have reached a level of saturation for these topics from the perspective of individual interviewees, they remain areas requiring further study and critical reflection.

On that point, though interviewees’ insights were generally supported by existing literature, additional reflections are necessary concerning the specifics. Regarding the concern about geographical underrepresentation in datasets, for instance, it should be recognised that representation from developing countries in global datasets has improved over the last decades due to the globalisation of clinical research, with some countries, such as South Africa and Brazil, participating in key projects of the clinical trials industry [26–28]. That being said, in general, it remains true that the process is mostly exclusive to large developing countries with large patient populations and inappropriate digital infrastructures. Indeed, the field of targeted therapies is still witnessing the migration of data from paper-based formats to digital formats, a process that tends to be less advanced in LMICs. Moreover, data fragmentation of the pharma and biotech industries adds to this problem, as it prevents the formation of large global data repositories. The situation of AI training datasets, in a way, reminds one of clinical trials in the 1980s, when campaigners asked for the diversification of patient populations from which data was collected [29]. Diversification poses its own risks for AI, however, as data from genetically diverse populations may turn into “unexpected data” in the workings of biomanufacturing algorithms. Hence, it will be important to the future of targeted medicines that algorithms “be tested for how they react when presented with unexpected data” [30].

It was also suggested by some interviewees that AI might reduce costs (and thus the biases associated with affluence). This resonates with similar arguments found in the literature that automation and AI can lead to more affordable cell and gene therapies [31]. It is known that manufacturing costs have a substantial impact on a therapy’s final prices. It is important to note, however, that AI might also heighten these costs. AI algorithms for biomanufacturing will be highly data intensive. Companies and organisations need access to large training datasets if they are to design robust and reliable AI-based software. In their study of AI-based diagnostic imaging algorithms, Larson et al. [30] noted, for instance, that AI products need to be constantly updated and monitored, a requisite that is being incorporated by regulatory frameworks. The same requirement is likely to be adopted by regulators of AI-based biomanufacturing systems in the future, which could heighten the costs of production, and may exclude middle-sized companies, including those in developing countries. If biomanufacturing becomes further centralised, it is not clear what the financial impacts of such changes will be on the industry. A more long-term view invites us to ask, therefore, to what extent the concentration of innovation in the hands of a few resourceful companies may hamper access and equity.

Regarding the self-correcting potential of routine AI data collection, some cutting-edge systems for automated manufacturing of cell and gene therapies have already incorporated AI for quality control and the real-time adjustment of manufacturing parameters [13, 14]. From this point of view, the accumulation of data, as well as the gradual enhancement of manufacturing algorithms, might be considered as important strategies to rid biomanufacturing of impending risks, imprecisions, and biases. Though there are reasons to be sceptical of technical solutions to bias, as several of our interviewees warned, one should not dismiss AI solutions altogether, as there may be ways in which it can still play a role in mitigating certain types of bias, whether technological or psychological [32].

Regarding transparency of bias reporting, there are promising examples of standards being set to understand unrepresentative datasets and their responsible use in research [33]. With the adoption of point-of-care manufacture (that is, the production of therapies in clinical settings), this idea becomes particularly pressing. Given the various technical and institutional prerequisites for point-of-care manufacture [34, 35], it is expected that, at least in the early stages, a limited number of hospitals will be performing and collecting data about biomanufacturing systems. Therefore, AI training datasets will be initially related to a few hospitals and countries. It remains to be understood to what extent such circumstances might impact on the performance of automated manufacturing systems when they are eventually taken to a larger number of hospitals and countries. Being transparent about data collection practices and their limitations will be crucial for better understanding the risks of bias as processes are made scalable.

Regarding the claims made by one of the interviewees that there may be low risk of bias in supply chain optimisations due to it concerning non-population data, recent trends in biomanufacturing problematize this idea. For example, point-of-care manufacture can be a viable solution for cell and gene therapies, which may require very quick manufacturing processes due to their short shelf life [34, 35]. This scheme is especially promising for autologous therapies, which are produced with cells and tissues collected from the body of people for whom the medicine is manufactured. In these point-of-care conditions, data is likely to become “hybrid data,” encompassing information regarding the patient, their medical history, the quality of the starting materials, and so on. Consequently, electronic patient records become indirectly (and perhaps, at some point, directly) connected to manufacturing execution software packages, blurring the frontiers between healthcare and therapy manufacture.

Finally, on the point of corrective bias, further elaboration and critique is required. As Kelly notes, biases are “deeply implicated in paradigmatic modes of knowledge acquisition, including sense perception, inductive reasoning, language learning, and scientific inquiry” [36]. If one takes the point seriously, then the issues is not, as Pot, Kieusseyan, and Prainsack [16] note, how to get rid of them, which would be impossible, but how to discern equitable biases from inequitable ones, or truth promoting from error prone ones. The answer is not an easy one to be had. As has been argued, there is no necessary link between bias and knowledge or equity [18, 36]. Some biases may be error prone, others truth preserving; some may lead to inequities, others indifferent to them or help address them. Noseworthy et al. [37], for instance, show how one biased medical AI system did not necessarily lead to biased outcomes. In that study, a deep learning system developed to “detect low left ventricular ejection fraction” from ECG data was trained on a “homogeneous” dataset (majority non-Hispanic white) but was just as effective for demographics not found in the training dataset. Related examples can also be found in the pharma industry, which often focuses on cancer and rare diseases to the detriment of other conditions. This could be said to be a bias, but one with positive results, insofar as it leads to treatment for certain populations. The point is even more pertinent when dealing with exceptional or rare cases (like the conditions which can be treated with cell and gene therapies), where “biases” can have positive outcomes for groups who otherwise would have few opportunities for treatment.

Even though it is right to reserve a place for corrective biases in medical AI ethics, then, one should also not treat that idea uncritically. If there is a disjunct between the intentions motivating corrective biases and any outcomes from it, even putative corrective biases could end up enhancing inequalities. Hence, although it has been argued that increasing focus (creating a bias) on underrepresented groups for a service for which they have been underserved (such as access to treatment or diagnosis) could help to create balanced outcomes [16], whether it does or not is an open question and one requiring further examination.

A next step in discriminating between biases, therefore, is to develop an empirical understanding of them and subject them to critical reflection and normative evaluation on a case-by-case basis. AI driven targeted medicines manufacturing lacks, however, both a descriptive and normative theory of bias. Given uncertainty over how digitalised targeted medicines manufacture will grow, one recommendation for developing that understanding would be to take an iterative horizon scanning of the technologies likely to be developed over the next five to ten years in order to reflect on their potential ethical challenges. This is because it is unclear what digital approaches will be deployed for targeted medicines manufacturing in the next decade. The field is in a developmental phase regarding digitalisation and the rapid change of AI research compounds the uncertainty. Moreover, our study is based on a small subsection of experts working in AI, bias, and targeted medicines, and is therefore limited to what they can reasonably imagine. An iterative horizon scanning approach would enable researchers to better anticipate the kinds of technologies that might be developed for AI-driven targeted medicines along with their potential ethical challenges vis-à-vis bias and to develop risk impact assessments based on that. A second approach would also be to unpack further the concept of corrective bias vis-à-vis health inequalities. Though it is beyond the scope of the paper to do that, the point made by our interviewees provides a first provocation in that direction.

## Conclusion

Our exploratory study showed multiple concerns about the possibility of bias in AI driven targeted medicines manufacturing. The most salient issue mentioned was social bias and how it might impact AI via unrepresentative datasets, with demographic, geographic and financial biases being singled out as particularly noteworthy. In certain areas, bias risks were less certain, and the riskiness was largely dependent upon whether AI relied upon population health data or not. However, respondents also noted how bias may have corrective value in terms of promoting health equity. Viewing bias primarily in terms of its negative impacts may cause confusion when bias is also seen a necessary part of the research process, as often happens when developing targeted medicines. Here corrective biases can provide an important counterpoint, by highlighting the important function certain types of bias may have in helping to address health inequalities. That said, corrective biases should not be taken at face value. Insofar as there is no necessary link between bias and values like truth or equity, they too should be subject to critical reflection. An important next step for the future of targeted medicines, therefore, is to develop a descriptive and normative account of bias to better understand the future pathways of bias and whether and how to mitigate it.

## Electronic supplementary material

Below is the link to the electronic supplementary material.

Supplementary Material 1

## Data Availability

The datasets generated and/or analysed during the current study are not publicly available due to privacy concerns.

## Footnotes

1 Sample size differs from the number of expertise areas represented as interviewees had expertise in more than one area.

## Abbreviations

AI: Artificial Intelligence
DNA: Deoxyribonucleic Acid
LMICs: Low–and Middle–Income Countries
NHS: National Health Service
NICE: National Institute for health and Care Excellence
UK: United Kingdom
(CAR) T cell: Chimeric Antigen receptor T cells
PPI: Patient and public Involvement
ECG: Electrocardiogram
