# Supplementary material for: Exploring bias risks in artificial intelligence and targeted medicines manufacturing
Source: BMC Med Ethics. 2024 Oct 17;25:113. doi: 10.1186/s12910-024-01112-1 (PMC11483979; doi:10.1186/s12910-024-01112-1)
Supplement: Supplementary file 1 — Supplementary Material 1 [file 12910_2024_1112_MOESM1_ESM.pdf]

Semi-structured interview guide

A) Interviewer background:

Can you tell me your age, position, role?

B) Topic Preliminaries

What is meant by precision medicine?

What kind of technologies are we dealing with?

How is the FTHM hub transforming targeted medicines?

C) Manufacturing Preliminaries

What is meant by “manufacturing” in precision medicine?

How does medicine manufacture traditionally work for precision medicines?

How is medicine manufacturing changing?

How is digital/AI impacting biological medicine manufacture?

D) Digital and AI

What are the most promising digital technologies for medicine manufacture?

What are the most promising AI technologies for medicine manufacture?

E) General ethical questions

What are your ethical concerns about precision medicine?

What are your ethical concerns about AI / digital technologies in precision medicine?

F) Bias

What do you understand by the term “bias”?

Do you see bias as arising in your work or one of the above technologies?

Why is bias a problem?

If so, what are the apparent causes?

How might one address it?

G) Follow up questions

Is there anything I've forgotten to ask that you’d like to have talked about?

Who else do you recommend I speak to about the above issues?

Is it ok to contact you with further questions?

Thank them for their time.
